# Interventionelle Radiologie – Ausbildung und Aufstiegschancen

**DOI:** 10.1007/s00117-024-01382-1

**Published:** 2024-12-09

**Authors:** Lisa Ullrich, Wibke Uller, Anne Frisch

**Affiliations:** 1 DIE Radiologie, Sonnenstraße 17, 80331 München, Deutschland; 2 Klinik für diagnostische und interventionelle Radiologie, Universitätsklinikum Freiburg, Medizinische Fakultät, Albert-Ludwigs-Universität Freiburg, Hugstetter Str. 55, 79106 Freiburg, Deutschland; 3 Klinik für Radiologie, Charité – Universitätsmedizin Berlin, Campus Virchow-Klinikum, Augustenburger Platz 1, 13353 Berlin, Deutschland

Mit der rasant fortschreitenden Entwicklung von Unterstützungssystemen der künstlichen Intelligenz (KI) sehen wir uns in der Fachdisziplin der Radiologie zunehmend mit der Frage konfrontiert, ob in diesem Berufszweig das ärztliche Personal in Zukunft durch Technik und KI ersetzbar sei. Bereits für die diagnostische Radiologie ist dies zu verneinen [1, 2] und für die Interventionsradiologie (IR) und interventionelle Neuroradiologie umso weniger zutreffend – menschliche manuelle Fertigkeiten sowie medizinisches Know-how sind in diesem Teilgebiet der Radiologie bislang keineswegs durch Maschinen ersetzbar [3]. Gleichzeitig hat sich die IR als integraler Bestandteil der modernen Versorgung von Patientinnen und Patienten etabliert, und die Nachfrage nach versiertem Fachpersonal steigt [4, 5], sodass sich wertvolle zukunftssichere Weiterbildungs- und Aufstiegschancen auf dem Gebiet der IR bieten.

## Die interventionelle Radiologie – vielseitig und von großem Bedarf

Die IR (inklusive der interventionellen Neuroradiologie) beinhaltet hierzulande ein schnell wachsendes Teilgebiet des Fachs Radiologie: Sie schließt die bildgestützte invasive Diagnostik sowie minimal-invasive Techniken zur Therapie zahlreicher Erkrankungen ein. Gefäßeröffnende Verfahren bei der peripheren arteriellen Verschlusskrankheit oder dem Schlaganfall und auch gefäßverschließende Verfahren bei Blutungen oder Aneurysmen gehören gleichermaßen zum Spektrum, wie die bildgestützte Gewinnung von Gewebeproben und die Drainage von Abszessen. Im Rahmen der Behandlung onkologischer Erkrankungen umfasst sie u. a. die ambulante Implantation von zentralvenösen Gefäßzugängen, die transarterielle Radio- oder Chemoembolisation und perkutane Ablationen. Die verwendeten Modalitäten für eine hochpräzise Steuerung der Eingriffe reichen von der Magnetresonanztomographie (MRT) über Computertomographie (CT) bis zur Sonographie. Allen interventionell-radiologischen Eingriffen ist dabei deren minimal-invasiver Charakter gemein – sprich, die Eingriffe werden häufig lediglich in Lokalanästhesie und ohne größere Inzisionen durchgeführt. Narkoserisiken, Blutverluste und Infektionsrisiken werden somit minimiert und die Dauer des Krankenhausaufenthalts sowie die Erholungsphasen nach den Prozeduren sind in der Regel kürzer. Durch die resultierenden Möglichkeiten der ressourcensparenden und komplikationsarmen Diagnostik und Behandlung von Patientinnen und Patienten steigt der Stellenwert interventionell-radiologischer Verfahren – sowohl im elektiven als auch im akuten Setting – zunehmend [4, 6].

## Der Platz im Studium und der fachärztlichen Weiterbildung

**Abb. 1:**
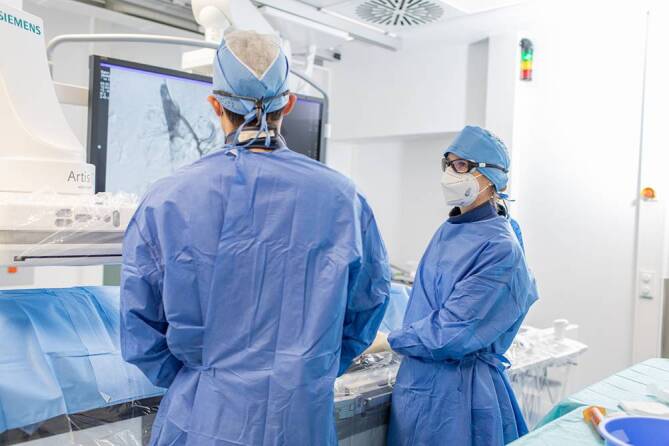
Supervidierende (*rechts*) bei der Assistenz einer Intervention. (Mit freundl. Genehmigung von Wibke Uller)

Die IR ist momentan während des Studiums der Humanmedizin und in den geläufigen nationalen medizinischen Curricula lediglich indirekt in die Themenfelder der minimal-invasiven Therapieformen subsumiert und/oder oftmals innerhalb der radiologischen Unterrichtseinheiten adressiert. Um das Interesse von Studierenden zu wecken und die Sichtbarkeit des Fachs zu erhöhen, sind gesonderte Maßnahmen, wie Hospitationen, Praktika oder zusätzliche Lehrangebote gewinnbringend [7, 8]. Auch nach Abschluss des Studiums ist in Deutschland der interventionelle Teilbereich in die radiologische fachärztliche Weiterbildung und in die Schwerpunktsetzung zur Neuroradiologie integriert. Während ihrer 5‑jährigen Weiterbildungszeit verbringen Assistenzärztinnen und -ärzte planmäßig einige Monate in der IR, um die von der Ärztekammer geforderten Eingriffszahlen für die Facharztreife zu erlangen. In der Rotation assistieren sie bei angiographischen oder CT-gesteuerten Eingriffen und/oder führen einzelne Schritte dieser unter Anleitung durch (Abb. 1). Bei weiterem Interesse kann mit Eigeninitiative eine Subspezialisierung im Rahmen von Zertifizierungsprogrammen der Fachgesellschaften nach der fachärztlichen Weiterbildung verfolgt werden. Ein gesonderter, durch die Ärztekammer geprüfter Schwerpunkt im Fach Interventionsradiologie existiert derzeit in Deutschland – im Gegensatz zu den anerkannten Schwerpunkten „Kinderradiologie“ und „Neuroradiologie“ – nicht.

Obwohl nationale Vorgaben der radiologischen Weiterbildung theoretisch anderes vorsehen und die Infrastruktur der interventionell-radiologischen Abteilungen besteht, wiesen sowohl nationale als auch internationale Umfragen von Weiterbildungsassistierenden auf ein Verbesserungspotenzial in der Ausgestaltung der interventionell-radiologischen Weiterbildung hin. Diese zeigt sich laut der Umfrageergebnisse mitunter in die Zeit nach der fachärztlichen Prüfung verlagert, von kurzer, unterbrochener oder ungeregelter Dauer und mit unzureichender Struktur und Supervision [9–11]. Weitere in Umfragen genannte Gründe, der Subspezialisierung der Interventionsradiologie nicht nachzugehen, umfassen u. a. die Strahlenexposition, die körperliche Belastung, die Arbeitsbedingungen und der Kontakt zu Patientinnen und Patienten [9, 10]. Das Argument der Strahlenbelastung verliert unter den zunehmend möglichen Schutzmaßnahmen und den geltenden gesetzlichen Bestimmungen fortschreitend an Gewicht. Eine Abfrage der Arbeitszeiten im Vergleich von diagnostischer und interventioneller Radiologie ergab keinen relevanten Unterschied bezüglich der Überstunden und wöchentlichen Arbeitszeit im klinischen Setting [10]. Aspekte des Kontakts zu Patientinnen und Patienten sowie der körperlichen Betätigung unterliegen großer Ambivalenz bei den Befragten und wirken für einige Weiterbildungsassistierende wiederum gerade vorteilhaft und profilerweiternd für das Fach Radiologie.

## Nutzung und Förderung des (Nachwuchs‑)Potenzials

Da alles in allem klinisch die Nachfrage, strukturell die Gegebenheiten und auch institutionell die Vorgaben existieren, steht einer guten Weiterbildung des interessierten Nachwuchses in der Theorie nichts im Weg. Letztlich gilt es, bestehende Strukturen optimal zu nutzen, um erstens eine fundierte, praxisorientierte interventionell-radiologische Basisausbildung während der Weiterbildungszeit und zweitens eine effiziente Überleitung in die weitergehende Spezialisierung im unmittelbaren Anschluss an die Weiterbildungszeit zu ermöglichen.

Grundlegend bedarf es während der fachärztlichen Weiterbildung einer gesicherten interventionell-radiologischen Rotation von adäquater Dauer von mindestens 3–6 Monaten oder ggf. auch nach interessenbasierter Länge [10]. Daneben sind ein erleichterter Zugang zu Übungsmodellen, standortunabhängige Simulatortrainings sowie spezielle ergänzende Lernformate für die Vorbereitung und Unterstützung vor und während der Rotationszeit essenziell, um effektiv praxis- und patient:innenorientiert während des aktiven interventionell-radiologischen Einsatzes tätig zu sein. Die Umsetzbarkeit und der Lernerfolg durch online abgehaltene, durch die DeGIR initiierte (simulatorgestützte) Lernsituationen wurden während der Pandemie dokumentiert. Die vorbereitende praktische und theoretische Auseinandersetzung mit dem Fach erlaubt nachweislich eine effiziente Nutzung der Rotationszeit und den Benefit, in der Folge eine solide grundlegende Routine zu entwickeln [12–15]. Von großem Nutzen ist dabei das öffentlich existierende vielfältige Angebot aus verschiedenen Veranstaltungen, Kursen und Workshops zur fundierten Weiterbildung für an der Interventionsradiologie interessierte Ärztinnen und Ärzten (Tab. 1). Im deutschsprachigen Raum setzt sich die Deutsche Gesellschaft für Interventionelle Radiologie und minimal-invasive Therapie (DeGIR) nicht nur für die Förderung des Nachwuchses bereits ab früher Weiterbildung ein, sondern stellt auch versierte Ansprechpartner:innen für Fragen zur Karriereplanung.

**Tab. 1:** *Fort- und Weiterbildungslandschaft in der Interventionsradiologie*. Einige Veranstaltungen und Lernmedien beinhalten kostenpflichtige Bestandteile. Das eigenständige Informieren über die Konditionen ist angeraten

Titel	Inhalt	URL
**Fachgesellschaft und Gremien**		
Deutsche Gesellschaft für Interventionelle Radiologie und minimal-invasive Therapie (DeGIR)	Fachvertretung für alle interventionsradiologisch und minimal-invasiv tätigen Radiologinnen und Radiologen in der Deutschen Röntgengesellschaft (DRG)	https://degir.de/
Lenkungsgruppe Nachwuchsförderung und Frauen in der IR	Arbeitsgruppe der DeGIR zur Informationsverbreitung von berufspolitischen, klinischen und wissenschaftlichen Themen rund um die interventionsradiologische Karriere sowie zur Gewinnung von Interessent:innen am Fach und Förderung der Vernetzung	https://degir.de/ueber-uns/lenkungsgruppen/nachwuchsfoerderung-frauen-ir/
Lenkungsgruppe Fort- und Weiterbildung	Arbeitsgruppe zur Ausgestaltung der Fortbildungsveranstaltungen der DeGIR beim IROS und beim Deutschen Röntgenkongress	https://degir.de/ueber-uns/lenkungsgruppen/
Cardiovascular and Interventional Radiological Society of Europe (CIRSE)	Fachvertretung für alle interventionsradiologisch und minimal-invasiv tätigen Radiologinnen und Radiologen in Europa	https://www.cirse.org/
**Curricula und Zertifizierungsprogramme**		
Curriculum der DeGIR/DGNR	Diese Orientierungshilfe bildet die Inhalte in der fachlichen interventionell-radiologischen Spezialisierung ab	https://degir.de/fortbildung/curriculum-ir/
Zertifizierungsprogramm der DeGIR	Unter entsprechenden Voraussetzungen kann eine DeGIR- bzw. DeGIR/DGNR-Zertifizierung zur interventionell-radiologische Subspezialisierung abgeschlossen werden. Auf Seiten der DeGIR und DRG sind auf Qualität geprüfte Kurse und Zentren einsehbar	https://degir.de/zertifizierung/zertifizierung-personen/
Zertifizierungsprogramm EBIR der CIRSE	Die Zertifizierung der europäischen interventionell-radiologischen Fachgesellschaft endet im EBIR-Examen (European Board of Interventional Radiology). Geleitet durch ein Curriculum und entsprechende online On-demand-Webinare in der CIRSE Academy kann umfassendes Wissen zum Fach angeeignet werden	https://www.cirse.org/certification/ebir/
**Veranstaltungen und Kurse**		
Simulatorkurse der DeGIR	Hands-on-Kurse an Angiographiesimulatoren, bei denen fallbasiert Indikationen, Kontraindikationen, Durchführung und Komplikationen erlernt werden	https://degir.de/fortbildung/simulatorkurse/
Zertifizierungskurs der DeGIR	Die Zertifizierungskurse sind nach Stufen strukturiert und teils on demand abrufbar	https://academy.mevis.de/drg/choose_login/?next=/drg/courses/description/2617/
Flinke-Finger-Programm der DeGIR	Diese Simulatorkurse sind speziell für Weiterbildungsassistierende konzipiert und finden auf dem RöKo und IROS statt	https://degir.de/fortbildung/flinke-finger-programm/
Simulatorkurse der DGNR	Simulatorkurse der DGNR sind für neuroradiologische Zertifizierungen anrechenbar	https://www.dgnr.org/de-DE/494/hands-on-basis-inr
Mediathek der CIRSE	Die Webinare und Kongressbeiträge der CIRSE werden in der CIRSE Library gebündelt und stehen zum Abruf bereit	https://library.cirse.org/
Veranstaltungskalender der DRG	Über eine Filterfunktion kann die Suche nach interventionell-radiologischen Kursen eingeschränkt und von der DeGIR geprüfte Kurse angezeigt werden	https://www.drg.de/de-DE/178/veranstaltungskalender/
**Kongresse**		
Deutscher Röntgenkongress (RöKo)	Der jährlich stattfindende Kongress der DRG bietet ebenfalls ein interventionell-radiologisches Programm, inklusive Hands-on-Workshops	https://roentgenkongress.de/
Interventionell-Radiologisches Olbert Symposium (IROS)	Die gemeinsame Jahrestagung der Deutschen (DEGIR), Österreichischen (ÖGIR) und Schweizerischen (SSVIR) Gesellschaften für interventionelle Radiologie und minimal-invasive Therapie dient nicht nur der Vernetzung mit Expert:innen auf dem Gebiet der IR, sondern ist bekannt für die Ausrichtung der Vorträge auf die Fort- und Weiterbildung. Die Wissensvertiefung und die fachliche Diskussion stehen hier im Vordergrund	https://www.irosonline.org/
CIRSE Annual Congress	Der Jahreskongress der CIRSE ist ein interventionell-radiologischer Kongress in Europa mit vielseitigem Vortrags- und Workshopprogramm	https://cirsecongress.cirse.org/about/theannualcongress/
**Stipendienprogramme**		
Fellowship Grant Programme der CIRSE	Die CIRSE bietet ein Stipendienprogramm an über eine Fördersumme von 3000 € zur Unterstützung eines einmonatigen Aufenthalts in einer interventionsradiologischen Abteilung innereuropäisch außerhalb des eigenen Aufenthaltslands zur Erweiterung des interventionsradiologischen Horizonts	https://www.cirse.org/society/fellowship-grant-programme-2/

Um die bisherige Sichtbarkeit des Fachs über die existierenden, vorrangig auf Eigeninitiative hin durchgeführten Zertifizierungen der Fachgesellschaften hinaus zu erhöhen, sind spezielle Programme direkt vor oder nach Erlangung der Facharztreife zur klinikinternen gezielten weiterführenden Spezialisierung in der IR unterstützenswert. Dies ist insbesondere auch für eine effiziente und erfolgreiche Notfallversorgung essenziell und kann im Sinne eines internen Fellowships oder einer Realisierung des Fellowship-Programms der CIRSE erfolgen.

## Die Zukunft

Der Bedarf an der Disziplin selbst sowie an Personen, die diese beherrschen, existiert. Die Vorteile einer modernen Fachdisziplin werden in Gänze bedient. Minimal-Invasivität in der Versorgung von Patientinnen und Patienten, klinischer und wissenschaftlicher Abwechslungsreichtum in der fachärztlichen Weiterbildung sowie eine Nutzung der Technik ohne Bedenken zukünftig durch sie ersetzt zu werden, sind nur einige der vielfältigen Gründe, das Fach als Subspezialisierung zu wählen. Darauf basierend steht und fällt eine gelungene Nachwuchsgewinnung mit einer Weiterbildungsumgebung, in der alle interessierten Lernenden und Weiterbildenden willkommen sind, sowie gesicherte Rotationszeiten, strukturierte Curricula und eine verlässliche Supervision flächendeckend gelebt werden. Die Nachfrage am Fach besteht von klinischer und institutioneller Seite. Jetzt ist es an uns allen, diese auch auf Seiten des interessierten Nachwuchses zu generieren und ihr gerecht zu werden.
